# Switch of NAD Salvage to *de novo* Biosynthesis Sustains SIRT1-RelB-Dependent Inflammatory Tolerance

**DOI:** 10.3389/fimmu.2019.02358

**Published:** 2019-10-11

**Authors:** Jingpu Zhang, Jie Tao, Yun Ling, Feng Li, Xuewei Zhu, Li Xu, Mei Wang, Shuye Zhang, Charles E. McCall, Tie Fu Liu

**Affiliations:** ^1^Scientific Research Center, Shanghai Public Health Clinical Center, Fudan University, Shanghai, China; ^2^Department of Infection Diseases, Shanghai Public Health Clinical Center, Fudan University, Shanghai, China; ^3^Department of Critical Medicine, Shanghai Public Health Clinical Center, Fudan University, Shanghai, China; ^4^Molecular Medicine Section, Department of Internal Medicine, Wake Forest University School of Medicine, Winston-Salem, NC, United States

**Keywords:** inflammation, immune tolerance, NAD biosynthesis pathways, indoleamine 2, 3-dioxygenase, SIRT1-RelB axis

## Abstract

A typical inflammatory response sequentially progresses from pro-inflammatory, immune suppressive to inflammatory repairing phases. Although the physiological inflammatory response resolves in time, severe acute inflammation usually sustains immune tolerance and leads to high mortality, yet the underlying mechanism is not completely understood. Here, using the leukemia-derived THP-1 human monocytes, healthy and septic human peripheral blood mononuclear cells (PBMC), we report that endotoxin dose-dependent switch of nicotinamide adenine dinucleotide (NAD) biosynthesis pathways sustain immune tolerant status. Low dose endotoxin triggered nicotinamide phosphoribosyltransferase (NAMPT)-dependent NAD salvage activity to adapt pro-inflammation. In contrast, high dose endotoxin drove a shift of NAD synthesis pathway from early NAMPT-dependent NAD salvage to late indoleamine 2,3-dioxygenase-1 (IDO1)-dependent NAD *de novo* biosynthesis, leading to persistent immune suppression. This is resulted from the IDO1-dependent expansion of nuclear NAD pool and nuclear NAD-dependent prolongation of sirtuin1 (SIRT1)-directed epigenetics of immune tolerance. Inhibition of IDO1 activity predominantly decreased nuclear NAD level, which promoted sequential dissociations of immunosuppressive SIRT1 and RelB from the promoter of pro-inflammatory *TNF-*α gene and broke endotoxin tolerance. Thus, NAMPT-NAD-SIRT1 axis adapts pro-inflammation, but IDO1-NAD-SIRT1-RelB axis sustains endotoxin tolerance during acute inflammatory response. Remarkably, in contrast to the prevention of sepsis death of animal model by IDO1 inhibition before sepsis initiation, we demonstrated that the combination therapy of IDO1 inhibition by 1-methyl-D-tryptophan (1-MT) and tryptophan supplementation rather than 1-MT administration alone after sepsis onset rescued sepsis animals, highlighting the translational significance of tryptophan restoration in IDO1 targeting therapy of severe inflammatory diseases like sepsis.

## Introduction

Inflammatory response is evolutionarily conserved for physiological adaptation to environmental stresses including infection (1). The immune system senses stress levels and develops distinctly resolvable or refractory inflammatory phenotypes. Very low and moderate stresses differentially stimulate inflammatory priming (2) and reprogram inflammatory response from pro-inflammation, immune suppression to resolution that restores homeostasis and establishes immune memory (3, 4); whereas, high or “extreme” stresses sustain immune tolerance. The age-related severe community-acquired pneumonia, lobular pneumonia, and sepsis are clinical examples of inflammatory priming, resolvable, and refractory inflammation, respectively, particularly the prolonged immune tolerant response contributes to over 70% death of sepsis (5–7). The underlying mechanism of the distinct acute inflammatory phenotypes remains unclear, making it difficult to precisely target the immune response in severe inflammation like sepsis.

One typical feature of the inflammatory response is high energy consumption, which results in a dramatic change on the NAD/NADH redox ratio. NAD is a well-known cofactor for hydride-transfer enzymes that directly participate in metabolic pathways; it is also a substrate for NAD-consuming enzymes including poly (ADP-ribose) polymerases (PARPs), CD38, and NAD-dependent protein lysine deacetylases sirtuins (SIRT). All of them have important biological functions, and dysregulation of them is associated with various age-related diseases such as cancer, diabetes, and neurodegeneration (8–10). In particular, the changes of intracellular NAD/NADH ratio is dynamically sensed by sirtuin family members to epigenetically regulate inflammatory priming (11), inflammation adaptation (12), and resolution (13). Disturbance of NAD homeostasis by sustaining high cellular NAD levels exacerbates clinical outcomes of acute inflammatory sepsis (14), while the persistence of low cellular NAD contents primes chronic inflammatory disorders (15).

NAD-sirtuin functions are compartmentalized into cytosol, mitochondrial, and nuclear pools. Nuclear NAD-dependent SIRT1, 6 and 7 reprogram gene expression and DNA repairing and regulate cellular activities of aging, immunity and metabolism etc. (16). Since the activation of these regulatory pathways constitutively consumes NAD, it thus has to be fueled by specific biosynthetic routes including the salvage and kynurenine (KYN) pathways (17, 18), both of which actively regulate inflammatory response. Via the NAD salvage pathway, NAD catabolite nicotinamide (Nam) is converted to nicotinamide mononucleotide (NMN), the rate-limiting step catalyzed by nicotinamide phosphoribosyltransferase (NAMPT). NMN is then adenylated by nicotinamide mononucleotide adenylyltransferase to form NAD. *NAMPT* is readily induced in neutrophils and monocytes/macrophages by infections or inflammatory mediators like TNF-α, IL-1β, or endotoxin lipopolysaccharide (LPS) (19).

Distinct to the NAD salvage, the KYN pathway contributes to the synthesis of new NAD molecules using the essential amino acid tryptophan (Trp) as substrate, in which indoleamine-2, 3-dioxygenase (IDO) catalyzes the first and rate-limiting step that converts Trp into *N*-formyl-kynurenine. There are two isoforms of IDO in mice and humans, designated as IDO1 and IDO2, which possess overlapping and distinct immunoregulatory functions. *IDO2* has a restricted pattern of expression in liver, kidney and immune cells, and has low efficiency in Trp metabolism. Its deletion does not affect plasm Kyn level (20, 21); whereas, *IDO1* is broadly expressed in various type of tissues and has high efficiency in Trp metabolism (22). The overall outcomes of *IDO1-*dependent Trp catabolism are to suppress the proliferation of pathogens (23) and to produce a broad immune suppression in physiological and pathophysiological conditions such as pregnancy (24) and cancer immune evasion (25). *IDO1* can be particularly induced in antigen-presenting dendritic cells, macrophages, and B cells by a number of pro-inflammatory stimuli including LPS, bacterial DNA, type I/II interferons, and soluble cytokine receptors to sustain immune tolerance by depleting Trp to trigger amino-acid-sensing signal transduction pathways (26, 27). Sepsis-induced immune dysfunction/immunosuppression at the tolerant stage is closely associated with IDO1 activity. Non-survivors from sepsis have higher Kyn/Trp ratio than survivors (28). Blockade of IDO1 activity prior to sepsis initiation by either gene knockout or IDO1 specific inhibitor 1-methyl-D-tryptophan (1-MT) reduces mortality of sepsis animals (29, 30). Interestingly, although IDO1-NAD pathway plays an important role in sustaining tolerance, it is not required for tolerance induction (26, 31).

Despite the established association of NAD and its synthesis with different inflammatory phenotypes, how they fine-tune the inflammatory phase during severe inflammation response remains unclear. We have recently demonstrated that NAD-dependent SIRT4 deprograms immune tolerance of acute inflammatory response (10), here we report that the switch of NAD synthesis from NAMPT-dependent salvage to IDO1-dependent *de novo* neosynthesis sustained immune tolerance. Mechanistically, activation of NAD *de novo* synthesis fueled nuclear NAD pool, which prolonged SIRT1 and RelB repressors bound at the promoter of inflammatory gene. Translation of this finding may inform new mechanism-based precision treatment of acute inflammatory disorders like sepsis.

## Materials and Methods

### Primary Human Peripheral Blood Mononuclear Cell Modeling of Acute Inflammatory Response

Human venous blood samples of healthy volunteers or sepsis patients were collected according to the protocol approved by the Ethics Committee of Shanghai Public Health Clinical Center, Fudan University. Peripheral blood mononuclear cells (PBMC) were isolated by ficoll-hypaque density gradient centrifugation. To isolate monocytes, 20 μl of CD14 MicroBeads (Miltenyi Biotec, USA) per 10^7^ total PBMC were added into PBMC suspension and incubated for 15 min at 4°C. Cells were washed with PBS, resuspended in 1 ml of PBS buffer and passed through MACS column in the magnetic field of MACS Separator. After wash away the unlabeled cells with PBS, the MACS column was removed from the magnetic separator and the magnetically labeled monocytes in the column were collected by 2 washes with PBS. Both PBMCs and monocytes were cultured in RPMI 1640 culture medium supplemented with 100 units/ml penicillin, 100 μg/ml streptomycin, 2 mM L-glutamine, and 10% fetal bovine serum (HyClone, Logan, UT). PBMCs or primary monocytes were stimulated with either 1 or 100 ng/ml bacterial endotoxin lipopolysaccharide (LPS) (Escherichia coli 0111:B4, Sigma) for indicated times to detect subcellular NAD contents and gene expression.

### Human THP-1 Monocytic Cell Modeling of Acute Inflammatory Response

THP-1 is a human monocytic cell line derived from an acute monocytic leukemia patient. THP-1 cells were obtained from the American Type Culture Collection and were maintained in complete RPMI 1640 medium. 0.5 × 10^6^/ml cells were stimulated with 10 or 1,000 ng/ml of LPS for indicated times to generate inflammatory phases of initiation (0–8 h), immune suppression (tolerance) (24–48 h) or resolution (48–96 h) (12, 13). For the detection of endotoxin tolerance, THP-1 cells were primarily stimulated with LPS (1,000 ng/ml) for indicated times, cells were then washed with RPMI medium and subjected to a secondary LPS stimulation (1,000 ng/ml) for another 1 h. The tolerant cells were determined by the repression of *TNF-*α gene expression in response to the second LPS stimulation. In specified experiments, THP-1 cells were stimulated with Pam3CSK4 (1 μg/ml, Invitrogen) for indicated times to determine its immunotolerant potential by activation of TLR1/2.

### Animal Model of Polymicrobial Sepsis

The study of polymicrobial sepsis animal model was approved by the Ethics Committee of Shanghai Public Health Clinical Center, Fudan University. The mice (C57BL/6) at the age of 6–8 weeks were purchased from Shanghai Lingchang Biological Science and Technology (Shanghai, China) and were bred in animal facility at Shanghai Public Health Clinical Center. Sepsis was induced using a standard protocol of cecal ligation and puncture (CLP) as described previously (32). After ligation, cecum was perforated two times with a 22-gauge needle, contents were returned, and abdominal incision was closed in two layers (peritoneum and skin). Mice were subcutaneously given 1 ml of normal saline immediately after surgery. Mice were treated intraperitoneally with either 1-methyl-D-tryptopan (250 mg/Kg body weight, Sigma), or a mixture of 1-methyl-D-tryptophan (250 mg/Kg body weight) and L-tryptophan (500 mg/Kg body weight, Sigma) at 6 h after sepsis onset, and were monitored for 7 days to generate survival curve. The same treatment with normal saline served as the control group.

### Liquid Chromatography–Mass Spectrometry Analysis of Metabolites of NAD Biosynthesis Pathways

Acute inflammatory response was induced in human THP-1 monocytes via 1,000 ng/ml LPS stimulation for 0, 8, 24, 48, 72, or 96 h. Cells were washed twice with cold phosphate-buffered saline (PBS), snap freezing immediately and stored at −80°C. The metabolite profiles of the LPS-stimulated cells in quadruplicate were measured by Metabolon, Inc. (Durham, North Carolina, USA) using liquid chromatography–mass spectrometry according to the protocol detailed recently (33). Each sample contained 20 million viable cells.

### Preparation of Cytoplasmic and Nuclear Extracts

Cytoplasmic and nuclear extracts were prepared as described previously (34). In brief, cells (1 × 10^6^) were washed twice with cold PBS and cytoplasmic extracts were prepared by incubation of cell pellets in harvesting buffer [10 mmol/L HEPES (pH 7.9), 50 mmol/L NaCl, 0.5 mol/L sucrose, 0.1 mmol/L EDTA, 0.5% Triton-X 100, 1 mmol/L] for 5 min on ice followed by centrifugation. The supernatants were collected and were immediately stored at −80°C. The nuclei pellets were washed once with buffer A [10 mmol/L HEPES (pH 7.9), 10 mmol/L KCl, 0.1 mmol/L EDTA, and 0.1 mmol/L EGTA] and re-suspended in 2 × buffer C [10 mmol/L HEPES (pH 7.9), 500 mmol/L NaCl, 0.1 mmol/L EDTA, 0.1 mmol/L EGTA, and 0.1% Igepal (NP-40)] for 15 min on ice. After centrifugation, supernatants were collected as nuclear extracts and stored at −80°C until used for NAD detection.

### Colorimetric NAD Assays

NAD contents in cytoplasmic and nuclear extracts were detected using colorimetric assay kits (Biovision) according to manufacturer's instruction as described elsewhere (35). In brief, 10 microliters of cytoplasmic or nuclear samples in duplicates in a 96-well-plate were mixed with 40 μl NAD extracting buffer and incubated for 5 min at room temperature to convert NAD to NADH. The resultant NADH samples and NADH standards were mixed for 10 min with 100 μl of reaction mixture and 10 μl of NADH developer for another 1 h. The optical densities were read at 450 nm. Total NAD levels of unknown samples were calculated from the standard curve analyzed by Prism software (GraphPad Prism, version 6.0, GraphPad Software, San Diego, CA) and were normalized against protein levels.

### Quantitative Real-Time RT-PCR

Levels of human *TNF-*α, *NAMPT*, and *IDO1* mRNA were measured as described previously (12) by quantitative real-time RT-PCR using gene-specific TaqMan primer/probe sets of *TNF-*α (Hs00174128_m1), *NAMPT* (Hs00237184_m1), and *IDO1* (Hs00158027-m1) in an ABI prism 7000 Sequence Detection System (Applied Biosystems). *GAPDH* or β-*actin* mRNA was determined using TaqMan primer (Hs02786624_g1 and Hs01060665_g1, respectively) and was used for internal loading control.

### *IDO1* Gene-Specific Knockdown and Knockout

*IDO1* gene specific knockout THP-1 cells were generated using CRISPR-Cas9 technique. The forward gRNA sequence was 5′-CACCGTACCACATTGATGAAGACGT-3′ and the reverse gRNA sequence was 2: 3′-AAACACGTCTTCATCAATGTGGTAC-5′. The gRNAs were subcloned into lentiCRISPRv2 vector (Addgene, Cambridge, MA) and co-transfected into HEK-293T cells (American Type Culture Collection) together with lentivirus helper plasmids psPAX2 and pND2.G (Addgene, Cambridge, MA) using trans293T (Healthgene, Canada) according to the manufacturer's protocol. Virus-containing supernatants were collected 48 h and 72 h after transfection, respectively, filtered to eliminate cells and added into THP-1 cell culture in the presence of 8 μg/ml polybrene. After 72 h, the virus-infected cells were selected with 1 μg/ml puromycin for 7 days; puromycin resistant cells were adopted for experiments. LentiCRISPRv2 vector without *IDO1* gRNA sequence was used as control.

### Chromatin Immunoprecipitation (ChIP)

The dynamics of SIRT1 and RelB binding at *TNF-*α promoter was analyzed using a ChIP assay kit (Active Motif, Carlsbad, CA) as detailed previously (12). In brief, DNA-protein interactions were crosslinked by incubating 5 × 10^6^ cells with 1% formaldehyde for 10 min at room temperature. Cells were lysed in 300 μl of SDS lysis buffer and sonicated to shear DNA to an average fragment size of about 400–1,000 base pair. After centrifugation, 100 μl of supernatant were mixed with 900 μl of ChIP dilution buffer and precleared by incubation with 50 μl of 50% protein-A agarose slurry for 30 min at 4°C. Chromatin was immunoprecipitated for overnight at 4°C with 5 μg of antibodies (Santa Cruz Biotechnology) against SIRT1 (H-300) and RelB (C-19). Isotype-matched IgG served as a negative control. The precipitated DNA was analyzed by PCR using promoter-specific PCR primer pairs (Integrated DNA Technologies) covering the κB binding sites at proximal *TNF-*α promoter region. The Primer sequences were listed as follows: *TNF-*α κB3 (at −98) forward, 5′-TACCGCTTCCTCCAGATGAG-3′ and reverse, 5′-TGCTGGCTGGGTGTGCCAA-3′; *RelB* forward, 5′-CAGAGCAATGGTCAGCGACG-3′ and reverse, 5′-CACAGT CTGGTGGACGATCG-3′ encircling κB1 (at −247) and –κB2 (at −175) sites. Equal amounts of PCR products were run onto 1.8% agarose gel and scanned using a typhoon scanner (GE Healthcare).

### Immunofluorescence Microscopy

Cells were cytospun onto slides and were fixed with 4% paraformaldehyde (PFA) for 20 min. Cell membrane was penetrated by 0.3% Triton-X-100 in phosphate-buffered saline (PBS-T) for 1 h at room temperature. Cells were then incubated with primary antibodies (Santa Cruz biotechnology) of ribbit anti-SIRT1 (H-300, 1:20 in PBS-T) or rabbit anti-RelB (C-19, 1:50 in PBS-T) overnight at 4°C. After 3 washes with PBS-T, cells were incubated with secondary antibody (Jackson Immunoresearch) of rhodamine (TRITC)-conjugated goat anti-rabbit IgG (Cat# 111-025-003, 1:2,000 in PBS-T) for 1 h at room temperature and followed by nuclear counterstaining for 5 min with DAPI (5 μg/ml, Sigma). Slides were then mounted with glycerol and analyzed using Leica TCS SP5 microscope (Leica) with LAS AF Lite 4.0 image browser software.

### Western Blot Analysis

Protein levels were analyzed by western blot as described previously (35). In brief, fifty microgram of cell lysates were separated by SDS-PAGE electrophoresis and transferred to a polyvinylidene difluoride membrane (PerkinElmer Life Sciences). Blots were blocked with 5% milk-TBS for 1 h at room temperature and probed overnight at 4°C using primary antibodies against NAMPT (H-300, Santa Cruz), IDO1 (GTX54705,Genetex), and β-actin (4E8H3, Santa Cruz). Protein complexes were subsequently incubated for 1 h at room temperature with goat anti-mouse IgG (Cat# SA00001-1, Proteintech) or goat anti-rabbit IgG (Cat# 28925, Rockland) diluted at 1:2,000 in blocking buffer and then detected by Enhanced Chemiluminescence Plus (GE Healthcare). Protein bands were scanned and densitometry analysis was performed using ImageJ software.

### Statistical Analyses

Differences of immune metabolic changes between two related conditions were analyzed by Student “*t*” test using GraphPad Prism version 6 (San Diego, CA). *P*-values less than 0.05 were considered significant. For experiments with more than two groups, Prism's Two-way Analysis of Variance (ANOVA) was used with *post-hoc* analysis of across multiple means. Welch's two-sample *t*-test was used to identify metabolites that differed significantly between experimental groups. Exact *P*-values are shown. Data points are Mean ± SD of replicates.

## Results

### Validation of *TNF-α* Gene Expression as a Representative Biomarker of Inflammatory Status and *GAPDH* as Reference Control of Gene Transcription

The most commonly used biomarkers of inflammation statuses are the cytokines. The highly expression of pro-inflammatory mediators indicates a cytokine storm response, while the minimized expression of them in response to secondary stimulation after an initial exposure to the same stimulus designates an immune suppressive or endotoxin tolerant status (36, 37). Two groups of pro-inflammatory genes are classified based on their expression dynamics, i.e., the primary response genes, such as *TNF-*α and *IL-1*β, which are generally induced within 1 h of stimulation, and the significantly delayed secondary response genes, such as *IL-6* and *NOS2*, because of the requirement for new protein synthesis and chromatin remodeling at their promoters (38, 39). To validate a representative indicator of inflammatory switch, we compared the dynamics of *TNF-*α*, IL-1*β, and *IL-6* gene expression in response to the first and the second LPS stimulation in THP-1 cells. Clearly, *TNF-*α and *IL-1*β mRNAs were peaked at 1 h and were tolerant to the second LPS stimulation about 2–4 h after LPS stimulation. In contrast, *IL-6* expression was significantly delayed and was peaked at 24 h after LPS stimulation; however, similar to the *TNF-*α and *IL-1*β expression, *IL-6* expression was minimized at all tested time points in response to the second LPS stimulation (Supplementary Figure S1). Thus, the fast responder genes of *TNF-*α or *IL-1*β can be the ideal indicator to timely reflect the inflammatory status.

Although housekeeping genes *glyceraldehyde-3-phosphate dehydrogenase* (*GAPDH*) and β*-actin* are universally used as reference controls for gene transcriptional and protein translational assays, *GAPDH* has been doubted as an ideal reference gene because it was reported to play roles in regulating inflammation. However, recent studies have demonstrated that *GAPDH* was steadily expressed in both the initial pro-inflammatory phase and the tolerant phase of acute inflammatory response (40, 41). Moreover, we validated *GAPDH* as a suitable reference control by showing that both *GAPDH* and β*-actin* genes were steadily expressed in THP-1 cell model of acute inflammatory response (Supplementary Figure S2). Therefore, in consistent with our previous studies (12, 13, 35, 42), we used the expression levels of *TNF-a* as the marker of inflammatory switch and *GAPDH* genes as reference control for gene expression assay in this study.

### Inflammatory Phenotypes Differentially Activate NAD Synthesis Pathways

To mimic the resolvable and tolerant inflammatory phenotypes, we firstly modeled lipopolysaccharide (LPS)-stimulated THP-1 human monocytes *in vitro*, which has been faithful to reprogramming acute inflammation epigenetics and post-translational NAD-dependent SIRT 1, 3, 4, and 6 pathways in human and mouse monocytes (12, 13, 35, 42). THP-1 cells were stimulated for 0–72 h with low (10 ng/ml) or high (1,000 ng/ml) doses of LPS. *TNF-*α gene expression was set as the indicator for inflammatory status. Both low and high doses of LPS induced peak *TNF–*α expression at 1 h, but high dose of LPS generated 30-fold higher *TNF-*α mRNA than low dose did. Furthermore, *TNF-*α mRNA levels induced by low or high doses of LPS returned to the baseline after 4 h and sustained low till 72 h (Supplementary Figure S3A). The kinetics of inflammatory response resembles that in human primary monocytes from sepsis patients and healthy volunteers given intravenous endotoxin (43–45).

After establishing the time course of different LPS doses, we next examined whether LPS doses could induce endotoxin tolerance differentially. THP-1 cells were pre-stimulated with low or high dose of LPS for 1 h or 24 h, and cells were then re-stimulated with 1,000 ng/ml LPS for 1 h to compare *TNF-*α mRNA levels. All cells primarily stimulated for 1 h with different doses of LPS responded to LPS re-stimulation. However, only the pre-stimulation by low dose LPS yielded significantly higher *TNF-*α mRNA level, in contrast, pre-stimulation by high dose LPS significantly reduced *TNF-*α mRNA in response to the second LPS stimulation (Supplementary Figure S3B). Among the cells primarily stimulated for 24 h, only the high dose of LPS pre-stimulation led to no significant changes of *TNF-*α expression in response to the second LPS stimulation (Supplementary Figure S3C).

**Figure 1:**
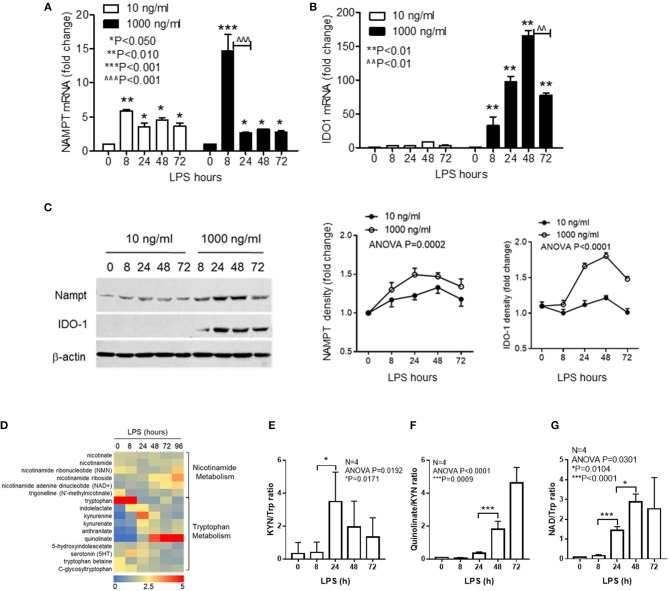
Inflammatory phenotypes differentially activate NAD synthesis pathways. THP-1 cells were stimulated for indicated times with either 10 or 1,000 ng/ml LPS, dynamic changes of *NAMPT* mRNA **(A)** or *IDO1* mRNA **(B)** were analyzed by qRT-PCR and protein levels of NAMPT and IDO1 were shown by western blot **(C)**. Time-dependent changes of protein levels were compared by densitometry using ImageJ software after normalizing against β-actin loading control. Data were shown as mean ± SD of three individual experiments. *P*-values were calculated by *post-hoc* comparisons between the treatment and control group unless otherwise specified with bars. **(D)** Variations of 15 different metabolites of nicotinamide and tryptophan metabolism were shown in the heatmap. THP-1 cells were stimulated with 1,000 ng/ml LPS for indicated times in triplicates in four individual experiments. Cells were snap-frozen and stored at −80°C and NAD metabolites were analyzed using liquid chromatography–mass spectrometry. The dynamics changes of ratios of KYN/Trp **(E)**, quinolinate/KYN **(F)**, and NAD/Trp **(G)** were shown as mean ± SD. *P*-values were calculated by *post-hoc* comparisons. KYN, kynurenine; Trp, tryptophan; NAD, nicotinamide adenine dinucleotide.

We previously reported that NAD-dependent epigenetics reprograms inflammation adaptation and high dose endotoxin activates NAMPT-dependent NAD salvage, which returns to baseline at early tolerant stage, whereas the NAD level continues to rise (12, 35). We thus reasoned that high dose rather than low dose LPS might activate IDO1-dependent *de novo* NAD synthesis to sustain immune tolerance. To test this possibility, cells were stimulated with either low dose (10 ng/ml) or high dose (1,000 ng/ml) LPS for indicated times, and the transcriptional levels of *NAMPT* and *IDO1* genes were compared by qRT-PCR. We found that low dose LPS induced a modest but significant increase in *NAMPT* transcription at 8 h with no significant change of *IDO1* gene expression. In contrast, high dose LPS stimulated substantial increases in both *NAMPT* and *IDO1* with different peak times: *NAMPT* gene expression peaked at 8 h, whereas *IDO1* expression peaked at 48 h when *NAMPT* expression re-approached baseline (Figures 1A,B). In support of gene expression dynamics, low dose LPS slightly increased NAMPT protein level without affecting IDO1 protein level, this contrasted with the increase in IDO1 protein level through 24–72 h after high dose of LPS stimulation (Figure 1C).

To further test if *IDO1* induction by high dose LPS could activate NAD synthesis and modify biochemistry profiles of NAD metabolism, we next semi-quantified the nicotinamide and tryptophan catabolism in response to high dose LPS stimulation using liquid chromatography–mass spectrometry. THP-1 cells were stimulated with 1,000 ng/ml LPS for indicated times, and the dynamic changes of 15 different metabolites of nicotinamide and tryptophan catabolism were shown in the heat map (Figure 1D). Kynurenine to tryptophan (KYN/Trp) ratio as the indicator of IDO activation was significantly increased at 24 h (Figure 1E), the downstream quinolinate to kynurenine ratio was significantly increased with the decrease of KYN/Trp ratio at 48 h after LPS stimulation (Figure 1F). Notably, NAD to Trp ratio was increased steadily during the course with high dose of LPS stimulation (Figure 1G).

LPS is a typical TLR4 agonist and is universally used in studies of acute inflammatory response. In order to test if other TLR agonists also potentially induce similar patterns of immune tolerance and *NAMPT* and *IDO1* gene expression as LPS did, we stimulated THP-1 cells with Pam3CSK4, a TLR1/2 agonist that signals to activate NF-kB pathway (46), and compared *TNF-*α*, NAMPT*, and *IDO1* mRNA levels. As similar as observed in LPS stimulated cells, the primary stimulation for 1 h by Pam3CSK4 (1 μg/ml) triggered peak expression of *TNF-*α, which was decreased to baseline at 8 h and remained low at 24 h after Pam3CSK4 stimulation. Pam3CSK4 pre-stimulation for either 1 h, 8 h or 24 h completely repressed *TNF-*α expression in response to Pam3CSK4 re-stimulation (Supplementary Figure S4A); Pam3CSK4 stimulation for 8 h induced a peak expression of *NAMPT*, which was returned to baseline level at 24 h (Supplementary Figure S4B), whereas, *IDO1* mRNA was gradually increased and reached its peak expression at 24 h after Pam3CSK4 stimulation (Supplementary Figure S4C). Thus, the highly activation of NF-kB pathway by either TLR1/2 agonist or TLR4 agonist switches NAD synthesis pathways and induces immunotolerance.

Taken together, these observations suggested that the stress level dominated the shift of NAD synthesis pathways and high dose of LPS sequentially activated NAD salvage and NAD *de novo* synthesis pathways during the development of immune tolerance.

### Chronic Inhibition of IDO1 Activity Minimizes Cellular NAD Content and Prevents Persistent Endotoxin Tolerance in Response to High Dose of LPS Stress

**Figure 2:**
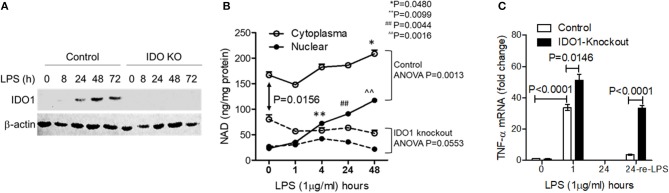
Chronic inhibition of IDO1 activity minimizes cellular NAD content and prevents persistent endotoxin tolerance in response to high dose of LPS stress. *IDO1* gene in THP-1 cells were gene-specifically knocked-out using CRISPR-Cas9 strategy. **(A)** *IDO1* gene knockout precluded IDO1 protein expression in response to high dose LPS stimulation. **(B)** Control or *IDO1* gene knockout THP-1 cells were stimulated with 1,000 ng/ml LPS for indicated times. Dynamic changes of NAD levels in cytoplasmic (the mixture of cytosol and mitochondrial NAD pools) and nuclear extracts were analyzed using colorimetric assay kits and normalized against protein contents. **(C)** Control or *IDO1* gene knockout THP-1 cells were stimulated with 1,000 ng/ml LPS for either 1 or 24 h, and a replicate of cells at 24 h was re-stimulated with 1,000 ng/ml LPS for 1 h, *TNF-*α mRNA was evaluated using qRT-PCR. Data in **(B,C)** were shown as mean ± SD of one of three individual experiments. *P*-values were to show the significant changes from baseline and were calculated by *post-hoc* comparisons.

To determine the importance of IDO1-dependent NAD biosynthesis in sustaining endotoxin tolerance status in response to high dose of LPS stress, we next studied the effects of *IDO1* gene knockout on changes of subcellular NAD levels and tolerance persistence in THP-1 cell stimulated by high dose of LPS. The expression of IDO1 protein was efficiently excluded using gene-specific CRISPR/Cas9 strategy (Figure 2A). Since nuclear NAD sensing controls immune tolerance response in sepsis models *in vitro* and *in vivo*, we assessed the dynamics of subcellular NAD pools in control and knockout THP-1 cells after high dose LPS stimulation. The nuclear NAD levels in control cells were significantly increased at 4, 24, and 48 h. In contrast, the extranuclear NAD levels, a mixture of cytosol and mitochondrial pools, did not change significantly until 48 h (Figure 2B). Surprisingly, *IDO1* gene knockout significantly reduced the basal cytoplasmic NAD, rather than the basal nuclear NAD levels when compared to those in control cells. However, *IDO1* knockout attenuated LPS-mediated increases in both nuclear and cytoplasmic NAD levels (Figure 2B).

To test if *IDO1* deficiency would attenuate the persistence of immune tolerance, we re-stimulated immune tolerant cells with high dose LPS and measured *TNF-*α transcriptional levels. As expected, *TNF-*α mRNA levels in both control and *IDO1* knockout cells were significantly increased at 1 h and were returned into baseline at 24 h (Figure 2C). Remarkably, control cells became tolerant after the pre-stimulation for 24 h, but *IDO1* knockout cells remained sensitive to LPS re-stimulation (Figure 2C), suggesting that IDO1-dependent NAD synthesis, particularly the resulting NAD increase in the nuclear, is essential to sustain immune tolerance.

### Acute Inhibition of IDO1 Activity Reduces Nuclear NAD Content and Breaks Endotoxin Tolerance in Response to High Dose of LPS Stress

**Figure 3:**
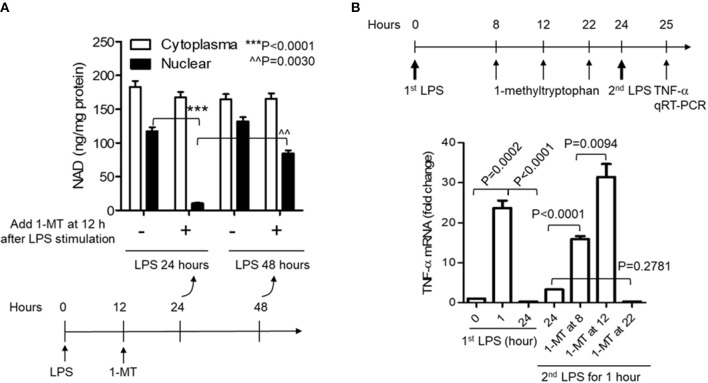
Acute inhibition of IDO1 activity preferentially reduces nuclear NAD content and breaks endotoxin tolerance during acute inflammatory response. **(A)** THP-1 cells were stimulated with 1,000 ng/ml LPS, and one replicate of cell cultures were added 1 mM of 1-methyl-D-tryptophan (1-MT) at 12 h after LPS stimulation. Cells were harvested either at 24 or 48 h after LPS stimulation, NAD levels in cytoplasmic and nuclear extracts were analyzed using colorimetric assay kits and were normalized against protein level. **(B)** THP-1 cells were stimulated with 1,000 ng/ml LPS, and one replicate of cell cultures were added 1 mM of 1-MT at 8, 12, or 22 h after LPS stimulation. Cell cultures were divided into two parts at 24 h, and one part of the cells was re-stimulated with 1,000 ng/ml LPS for 1 h. *TNF-*α mRNA was evaluated using qRT-PCR. *P*-values were calculated by *post-hoc* comparisons.

Reducing cellular NAD content by *IDO1* gene knockout prior to inflammatory triggering must disturb the natural course of acute inflammatory response and affects the evaluation of IDO1's contribution to immune tolerance persistence. Specific chemical compounds can temporarily and flexibly modulate IDO1 activity at any time point during the course of inflammatory response. We then assessed whether temporarily inhibiting IDO1 activity could reduce nuclear NAD and break immune tolerance. To do this, we first inhibited IDO1 activity in THP-1 monocytes using 1-methyl-D-tryptophan (1-MT) after the onset of LPS stimulation. When cells were treated with 1-MT (1 mM) at 12 h after LPS (1,000 ng/ml) stimulation, the time point that the immunometabolic co-switch occurs (12, 32, 35), the nuclear NAD was significantly decreased at 24 h, but was reversed at 48 h after LPS stimulation. Surprisingly, 1-MT administration did not induce any significant changes on the cytoplasmic NAD levels (Figure 3A).

To test if the acute reduction of nuclear NAD by 1-MT could break immune tolerance, 1-MT was added into THP-1 cell cultures at 8, 12, or 22 h and cells were harvested at 24 h after LPS stimulation, i.e., LPS stimulated-cells were treated for 16, 12, or 2 h with 1-MT, respectively, before their harvest at 24 h. THP-1 cells were then washed, and one part of the cultured cells was re-stimulated with 1,000 ng/ml LPS for another 1 h. *TNF-*α mRNA was then evaluated by qRT-PCR. As expected, the peak *TNF-*α mRNA level of control cells was induced at 1 h and was tolerant to LPS re-stimulation at 24 h. In contrast, 1-MT treatments either at 8 or 12 h after LPS stimulation significantly increased *TNF-*α mRNA levels in response to LPS re-stimulation. The 1-MT treatment at 12 h maximized the tolerance reversal than that observed at 8 h. However, 1-MT treatment at 22 h did not affect *TNF-*α expression in response to the second LPS stimulation. These observations suggested a ~12 h half-life (t_1/2_) of 1-MT physiologic efficiency (Figure 3B) and were aligned with the kinetics of 1-MT-dependent reduction of nuclear NAD levels (Figure 3A).

### IDO1 Activity Determines SIRT1-RelB Binding Status at *TNF-α* Promoter

**Figure 4:**
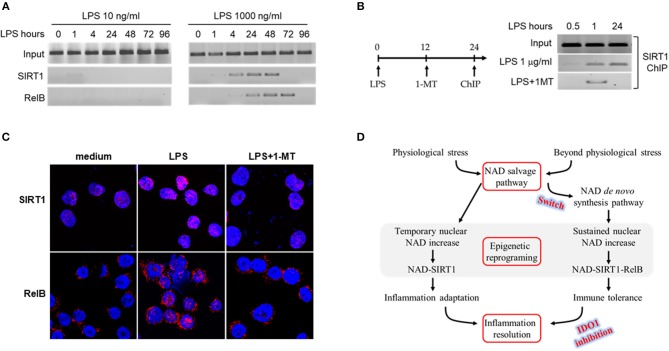
IDO1 activity determines SIRT1-RelB binding status at *TNF-*α promoter. **(A)** THP-1 cells were stimulated with either 10 or 1,000 ng/ml LPS for indicated times. The dynamics of SIRT1 or RelB binding in proximal region of *TNF-*α promoter were analyzed by ChIP using gene specific antibodies. **(B)** THP-1 cells were stimulated with 1,000 ng/ml of LPS for 0.5, 1, or 24 h, and one replicate of cell cultures was added 1 mM of 1-MT at 12 h after LPS stimulation. Cells were harvested and the promoter bound SIRT1 was analyzed by ChIP. **(C)** Dynamics of SIRT1 and RelB translocation between cytoplasm and nucleus in response to high dose LPS. THP-1 cells were stimulated with 1,000 ng/ml LPS for indicated times, and one replicate cell was treated with 1 mM 1-methyl-D-tryptophan at 12 h after LPS onset. Cells were then stained with rabbit antibodies against SIRT1 or RelB and the nuclei were counterstained with DAPI. Gene specific staining was imaged using Leica TCS SP5 microscope (Leica) with LAS AF Lite 4.0 image browser software. One of three individual experiments was shown. **(D)** Depiction of epigenetic reprogramming of low or high dose endotoxin-mediated inflammatory response. ChIP, chromatin immunoprecipitation.

We have reported that SIRT-dependent epigenetics reprograms immune tolerance in both cell and animal models, and blocking NAD-dependent activations of SIRT1 or 2 reverses immune tolerance, reactivates immune resistance and improves survival (32, 47). Epigenetic mapping of high dose LPS responses revealed that the interaction between SIRT1 and NFκB RelB at promoters of pro-inflammatory genes generates reversibly silent heterochromatin during the development of immune tolerance (12). Furthermore, blocking SIRT1 by its specific inhibitor EX-527 at immune tolerance stage dissociates RelB from *TNF-*α promoter and recruits acetylation-activated NFKB/p65 onto it, thereby liberating euchromatin for the reactivation of inflammatory genes in response to LPS (12). Since IDO1 directs immune tolerance and IDO1 activation is stress level-dependent (Figure 1), we reasoned that IDO1-dependent accumulation of nuclear NAD contributes to the epigenetic reprogramming of immune tolerance phenotype. To test this notion, we determined the dynamics of SIRT1 and RelB binding at *TNF-*α promoter in response to low (10 ng/ml) or high (1,000 ng/ml) dose endotoxin. Low dose LPS quickly stimulated traces of SIRT1 binding at *TNF-*α promoter, however, it quickly disassociated from the promoter 1 h after LPS stimulation; we did not detect RelB binding at *TNF-*α promoter at all tested time points (Figure 4A left panel). In contrast, high dose LPS induced both SIRT1 and RelB binding at *TNF-*α promoter. The dynamics of SIRT1 and RelB binding at *TNF-*α promoter were consistent with that of the nuclear NAD changes and the development of immune tolerance (Figure 3). SIRT1 and RelB were dissociated from *TNF-*α promoter at 48 and 72 h, respectively, after LPS stimulation (Figure 4A right panel). The temporal dissociation of SIRT1 and RelB from the *TNF-*α promoter in endotoxin tolerant cells was aligned with the restorative dynamics of *TNF-*α gene expression in response to endotoxin re-stimulation. Thus, the dynamics of SIRT1 and RelB dissociation from *TNF-*α promoter were paralleled with the dynamics of tolerance reversal and inflammation resolution.

As either chronically or acutely inhibition of IDO1 activity excludes immune tolerance (Figures 2, 3), we next studied the effect of IDO1 inhibition on the dynamics of SIRT1 binding at *TNF-*α promoter. THP-1 cells were pre-stimulated with LPS for 12 h, and were treated for another 12 h with the combination of LPS and 1-MT (1 mM). It was found that, upon LPS stimulation, SIRT1 was recruited at *TNF-*α promoter at 1 and 24 h; however, inhibiting IDO1 activity with 1-MT at 12 h dissociated SIRT1 from *TNF-*α promoter at 24 h (Figure 4B). In support of the inhibitory effect of 1-MT on tolerance deprogramming, we next monitored the dynamics of SIRT1 and RelB translocation between cytoplasm and nuclear by confocal analysis. THP-1 cells were stimulated with LPS (1,000 ng/ml) for 24 h, and 1–MT (1 mM) was added into one set of the cell culture at 12 h during the course of LPS stimulation. SIRT1 was exclusively stained in nuclear at the basal status and its staining density in nuclear was significantly increased at 24 h; however, SIRT1 induction by LPS was attenuated by 1-MT treatment (Figure 4C up panel). In contrast to the nuclear staining of SIRT1, RelB was exclusively stained in cytoplasm at the basal status and was presented in both cytoplasm and nuclear after LPS stimulation; 1-MT treatment precluded the translocation of cytoplasmic RelB to nuclear (Figure 4C bottom panel). The main findings from the THP-1 cell model are summarized in Figure 4D.

### The Switch of NAD Synthesis Pathways in Primary Peripheral Blood Mononuclear Cells (PBMC) in Response to High Dose of LPS Stress

To confirm if the data from THP-1 promonocytes can be duplicated in primary monocytes, the human CD14+ monocytes were isolated from PBMCs and stimulated with 1 μg/ml LPS for 0 h, 8 h or 24 h, the gene expression dynamics of *TNF-*α*, NAMPT*, and *IDO1* were analyzed by RT-PCR. Similar to that in THP-1 cells, *TNF-*α mRNA was marginally increased at 8 h and minimized at 24 h after LPS stimulation. *NAMPT* mRNA was peaked at 8 h and down-regulated at 24 h, in contrast, *IDO1* mRNA was gradually increased and peaked at 24 h after LPS stimulation (Supplementary Figure S5).

**Figure 5:**
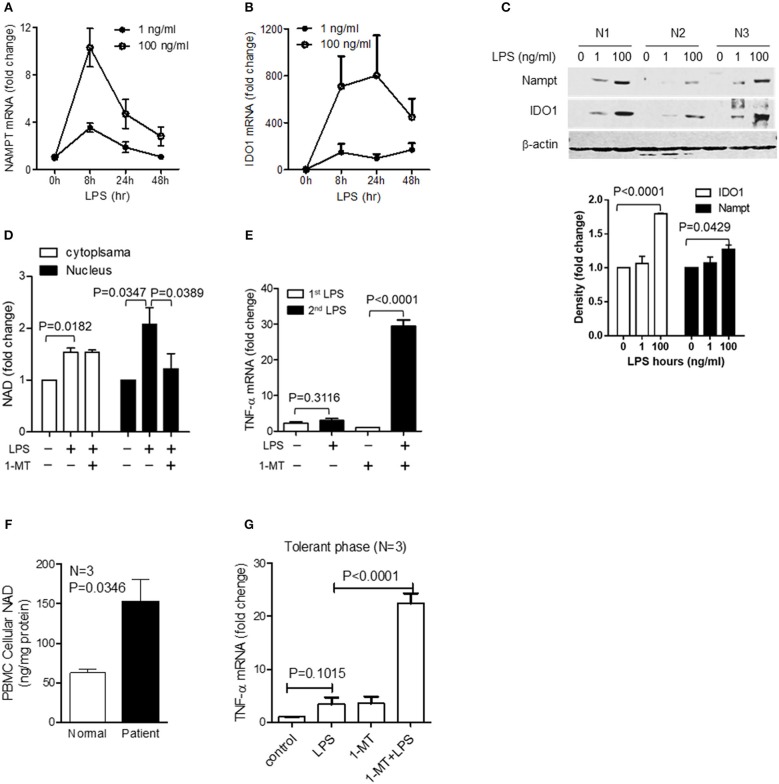
The switch of NAD synthesis pathways in primary peripheral blood mononuclear cells (PBMC) in response to high dose of LPS stress. Primary PBMCs of healthy volunteers were stimulated with either 1 or 100 ng/ml of LPS for indicated times. Dynamics of *NAMPT* **(A)** or *IDO1* **(B)** gene expression were analyzed by qRT-PCR, and the changes of corresponding protein levels were shown by western blot analysis and semi quantitatively analyzed by densitometry using ImageJ software **(C)**. **(D)** PBMCs were stimulated with 100 ng/ml LPS, and one replicate was added 1 mM of 1-MT at 12 h after LPS onset. Cells were harvested at 24 h after LPS stimulation. Cytoplasmic and nuclear NAD levels were detected using colorimetric assay kits and were normalized against the protein contents. **(E)** PBMCs were treated as in **(D)**, and one replicate of cells at 24 h was re-stimulated with 100 ng/ml LPS for 1 h, *TNF-*α mRNA was evaluated using qRT-PCR. Data were shown as mean ± SD of three healthy volunteers. **(F)** Basal cellular NAD levels of healthy and septic PBMCs was evaluated using colorimetric assay kits. **(G)** The tolerant status of septic PBMCs was determined by *TNF-*α gene transcription after LPS (100 ng/ml) stimulation for 1 h and the IDO1-dependent tolerance of septic PBMCs was determined by *TNF-*α gene transcription after treating cells with 1 mM1-MT for 12 h followed by LPS (100 ng/ml) stimulation for 1 h. Data were shown as mean ± SD of three healthy volunteers and three sepsis patients.

However, acute inflammation is an orchestral response of the innate and acquired immune cells, and we have previously proved that the LPS-mediated epigenetic, metabolic and energetic response in THP-1 cells can be reproduced in the immune cell mixture of human PBMCs and murine splenocytes (12, 13, 32, 35, 42). Others reported that endotoxin stimulation of the whole blood cells or post-mortem splenocytes of sepsis patients produces the similar cytokine profile as monocyte does (48, 49). Thus, to strengthen the potential translation of this study's findings, we used human primary PBMCs to assess the dynamics of gene transcription and protein translation of NAMPT and IDO1 and the NAD pool modifications in response to low (1 ng/ml) or high dose (100 ng/ml) of LPS. Similar to the observations in THP-1 cells, low dose LPS triggered suboptimal *NAMPT* and *IDO1* gene transcription and their corresponding protein translation. In contrast, high-dose LPS increased the mRNA and protein levels of both NAMPT and IDO1 (Figures 5A–C). Consistent with this dose- and time-dependent increase, 1-MT treatment significantly decreased nuclear NAD levels, instead of the extranuclear levels (Figure 5D). Moreover, confirming a predicted effect of IDO1 on immune tolerance, 1-MT treatment reverses high dose LPS-mediated tolerance (Figure 5E).

To further examine the potential for clinically relevant acute systemic inflammation from sepsis, we compared the basal NAD levels in PBMCs from normal humans and septic shock patients with immune tolerance. Basal NAD levels of septic PBMCs were significantly higher than those of normal PBMCs (Figure 5F). To test if inhibiting IDO1 activity would reverse immune tolerance of septic PBMCs, cells were pretreated for 12 h with 1 mM 1-MT and were stimulated by high dose endotoxin for 1 h. Tolerant status of septic PBMCs was demonstrated by minimal *TNF-*α transcription in response to high dose LPS stimulation. However, 1-MT treatment significantly increased *TNF-*α expression, indicating that 1-MT can break the immune tolerance of septic PBMCs (Figure 5G).

### Combination Therapy of IDO1 Inhibition and Tryptophan Supplementation Rescues Septic Animals

**Figure 6:**
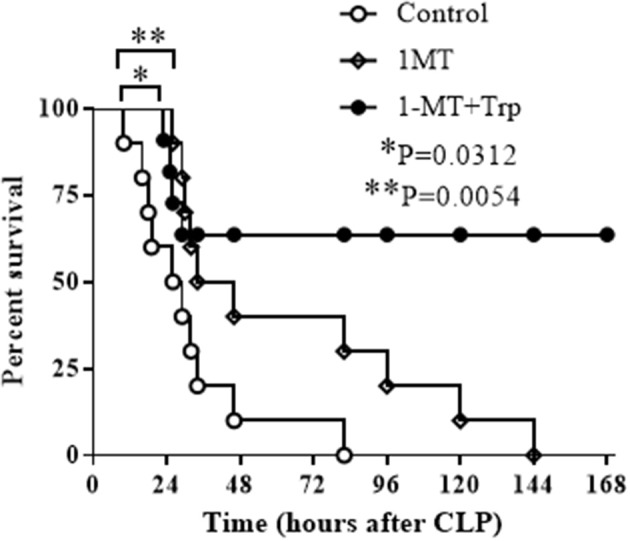
Combination therapy of IDO1 inhibition and tryptophan supplementation rescues septic animals. C57 BL/6 mice at age of 6–8 weeks were induced into polymicrobial sepsis by cecal ligation and puncture procedure. Six hours after sepsis onset, mice were intraperitoneally injected with normal saline (control group), 1-methyl-D-triptophan (250 mg/Kg body weight) or combination of 1-methyl-D-triptophan (250 mg/Kg body weight) and L-tryptophan (500 mg/Kg body weight). Animals were monitored for 7 days to plot survival curves. Each group contains 10 animals.

Previous studies have shown that the blockade of IDO1 activity prior to sepsis induction either by gene specific knockout or by 1-MT inhibition increases survival rate of septic animals (29, 30). To approach the clinical practice, we then tested if the inhibition of IDO1 after sepsis induction could also protect mice from sepsis death. C57 BL/6 mice at the age of 6–8 weeks were induced polymicrobial sepsis by cecal ligation and puncture (CLP) procedure, and were treated with either saline, 1-MT (250 mg/Kg body weight), or the combination of 1-MT and L-tryptophan (500 mg/Kg body weight) at 6 h after sepsis initiation. Mice were monitored for 7 days. To our surprise, although 1-MT treatment alone shifted the survival curve to the right, all animals died eventually. In contrast, the combination therapy of 1-MT and tryptophan rescued over 60% of septic animals (Figure 6).

## Discussion

This study explored NAD homeostasis as the molecular support of different inflammatory phenotypes in response to low or high dose of LPS stresses, which may direct the proceeding outcomes of severe inflammation.

Metabolic redox reactions reversibly reduce NAD into NADH, whereas PARPs and SIRTs irreversibly cleave NAD into nicotinamide and ADP-ribose (17, 18). In order to maintain NAD homeostasis and preserve cellular functions, mammalian cells evolutionally conserve NAMPT-dependent NAD salvage to acutely recycle nicotinamide to NAD and, if extreme response goes beyond the control of NAMPT pathway, IDO1-dependent neosynthesis would add new NAD molecules from tryptophan catabolism (17). Although the individual contributions of NAMPT or IDO1 to inflammation have been extensively discussed, the connections between these two pathways remain unclear. It appears that, similar to the sequential development from innate to acquired immunity, NAMPT-dependent salvage serves as the first line protector of NAD homeostasis, whereas IDO1 is induced to compensate the functional insufficiency of NAD salvage only if required under the context like severe inflammation. This notion is highlighted by the observations in our current and previous studies (12, 32) that controlled inflammation primarily activates NAD salvage path to silence pro-inflammatory genes and protects cells and organs from the cytotoxicity of cytokine storm. This protective adaptation of pro-inflammation is mediated by NAD-SIRT1-dependent epigenetics (12), and is beneficial to the timely resolution of inflammation by activating mitochondrial SIRT4, which counters SIRT1 actions (13). We have previously reported that NAMPT expression returned to the baseline upon the inflammatory resolution (12). The present study further demonstrated that extreme stress response activates the switch of NAD biosynthesis pathways from salvage to IDO1 path, and the latter pathway was found to particularly increase nuclear NAD level and sustain SIRT1-RelB-dependent tolerant epigenetics. Inhibiting IDO1 activity prior to sepsis initiation has been reported to improve the outcomes of septic animals (29, 30). These findings hint that a yet unknown physiological feedback mechanism would be responsible for IDO1 inhibition and the inflammation resolution in sepsis survivors, but it likely fails in sepsis non-survivors.

In addition, in contrast to acute inflammation, chronic inflammation reduces NAMPT activity and cellular NAD content (50), therefore, it can be inferred that the cells under the contexts of aging, obesity, and diabetes etc., which lose the first line defense of NAD homeostasis and NAD-dependent adaptation mechanism, might lead to persistent pro-inflammation. Accordingly, the comorbidities of advancing age and chronic inflammatory diseases usually prime severe pro-inflammation in response to infection due to the insufficiency of NAD-SIRT1-dependent epigenetic control, which deactivates NFκB/p65 at promoters of pro-inflammatory genes and silence cytokine storm response (12, 51). Therefore, the choice between resolvable inflammation and persistent tolerance appears to depend on if the switch of NAMPT- to IDO1-dependent NAD homeostasis occurs.

In particular, as the dynamics of nuclear NAD pool contributing to both NAMPT-dependent inflammation adaptation (12, 52) and IDO1-dependent tolerance persistence, the homeostasis of nuclear NAD pool becomes crucial in determining inflammatory phenotypes. Our current study and others' research (52) observed that either the activation of IDO1-dependent NAD neosynthesis or NAMPT-dependent NAD salvage increases the nuclear NAD contents, while the blockade of IDO1 or NAMPT with their specific inhibitors significantly reduced the nuclear NAD pool. The nuclear NAD dynamics controls the outcomes of inflammation by activating NAD-SIRT1 axis or NAD-SIRT1-RelB axis, both of which are immune repressors separately regulating the generation or persistence of immune tolerance (12). It was reported that exposure of macrophages to very low dose endotoxin diminished RelB protein level through interleukin receptor-associated kinase 1 and Tollip (Toll-interacting protein)-dependent mechanisms, and resulted in a hyper-pro-inflammatory response upon endotoxin re-stimulation (53); mediate dose of vaccine trained macrophages to increase NAD/NADH ratio through dectin-1–Akt–mTOR–HIF-1α pathway (3) and modified epigenetic profiles of histone proteins to generate immune memory (4); in contrast, high dose endotoxin stimulated increases in cellular NAD and sustained immune tolerance (12), and this is consistent with our observations in this study. Taken together, we can conclude that the differences in stress response induced by low to high endotoxin doses could switch NAMPT-dependent nuclear NAD homeostasis to IDO1-dependent nuclear NAD increases, thereby, shifting immune adaptation to persistent immune tolerance.

The consequences of nuclear NAD increases are to activate biological functions of the two major families of nuclear NAD sensors PARPs and sirtuins, which apparently act oppositely on inflammation regulation. PARPs metabolize NAD and catalyze protein ADP-ribosylation to potentiate the pro-inflammatory response by co-activating NFκB signal or by repressing the anti-inflammatory function of sirtuins in NAD-dependent or independent ways (54, 55), leading to the development of chronic inflammation and acute multiple organ failures of septic shock (56–58). In contrast, nuclear sirtuins consume NAD to catalyze deacetylation of histone and non-histone proteins, leading to the generation and persistence of immunosuppression status (12, 59–61). Upon inflammatory activation, SIRT1 firstly deacetylates non-histone transcription factor NFκB/p65 and functionally silences pro-inflammatory genes; it then deacetylates histone proteins, switches euchromatin to reversible heterochromatin and structurally represses inflammation (12). If extreme inflammation occurs, SIRT1 could further couple with NFκB/RelB to form immune repressor complex at the promoters of pro-inflammatory genes and sustain immune tolerant status (12). Thus, it appears that pro-inflammation triggers SIRT1-dependent functional silencing or a switch from the functional repression to chromatin structural silencing determines it resolvable or refractory. Similar to the SIRT1 action, the nuclear NAD sensor SIRT6 also exerts anti-inflammatory actions. It represses NFκB/p65 binding at target promoters and catalyzes deacetylation on histone H3K9 to form repressive heterochromatin (60–62). In addition to the direct action on the epigenetic reprogramming of inflammation, SIRT1 and 6 coordinate metabolic modifications to support immune suppression, in which SIRT1 deacetylates PGC-1 to activate β-oxidation, while SIRT6 represses HIF-1α target genes to shut off glycolysis (35, 61). The current study, together with our recent report (13), suggested that timely restoration of NAD homeostasis deactivates sirtuin-dependent epigenetic programming of inflammatory genes, reverses immune suppression and metabolic polarization, and resolves inflammation. However, how the reciprocal actions of the pro-inflammation promoter PARPs and immune suppressor sirtuins are coordinated remains unclear, but it should be associated with the dynamics of NAD homeostasis during the course of inflammatory response.

In contrast to the immediately significant change of nuclear NAD, we observed an unexpected dynamics of cytoplasmic NAD pool (primarily the mitochondrial NAD pool) which underwent a subtle change in the early phase but became significantly increased in the late phase. This challenges the well-clarified concept that immune response is an immunometabolic process and is regulated by the crosstalk of mitochondrial energetics and nuclear epigenetics (12, 35, 42, 50, 63, 64). The apparent uncoupling of mitochondrial NAD dynamics and inflammatory response was probably attributed to the large content of mitochondrial NAD pool, which represents up to 70% of total NAD content depending on cell type, and is about 40-fold more than cytosol and nuclear pools (17, 18). The large content of mitochondrial pool makes it insensitive to a small amount of NAD fluctuation until the changes of NAD level become significant. Instead, a smaller nuclear NAD pool becomes the highly sensitive center to sense the delicate NAD oscillation and trigger immediate response to signals related to NAD changes. This delicate design of NAD pool sizes makes the nuclear NAD pool preferential to the mitochondrial pool in controlling physiological activities like inflammation.

The clinical significance of sustained immunotolerance is particularly highlighted in severe sepsis, which is defined as an immunosuppressive disorder with high mortality (6). Because of this immune abnormality, severe sepsis response usually fails to eliminate the original infect agents and adequately respond to the second infections (65–67), making it a leading cause of sepsis death. Although the knowledge of immunosuppression under sepsis context are incomplete, two major molecular mechanisms have been defined, i.e., the T lymphocytes exhaustion with the increase in the ratio of T regulatory/T effect cells and the epigenetic reprogramming of pro-inflammatory gene expression (12, 68, 69). Both of them have been further confirmed by the mechanism-based immunostimulating therapies in clinical trials and experimental therapies (6, 32), which have achieved encouraging success in contrast to the failure of over 100 phase II and phase III trials of anti-inflammatory drugs that predominantly repress cytokine storm response (70, 71). For example, administration of IL-7 or GM-CSF at immunosuppression stage of sepsis patients restored immune cell homeostasis (72, 73); epigenetic targeting at immunosuppressive stage of septic mice by inhibiting SIRT1 activity promoted inflammatory resolution (32); all of which successfully reversed immune tolerance and significantly improved outcomes of human and animal sepsis. Thus, the immunomodulatory therapies against immunotolerance promise the effective approaches with a hope to save the lethal sepsis victims.

We demonstrated here that the IDO-dependent NAD biosynthesis contributes to the epigenetic mechanism of immune tolerance. IDO1-targeted therapies were proved to effectively protect septic animals from death by administration of IDO1 inhibitor 1-MT before sepsis initiation (29, 30). This protective effect was achieved by the mechanisms include (i) the increasing LPS-induced CXCL-2 and CXCL-1 production and the recruitment of neutrophils and mononuclear cells (29), (ii) sustaining physiological ratio of inflammatory to anti-inflammatory genes (30), and/or (iii) precluding the development of immune tolerance, and thus accordingly responding to subsequent infection. However, due to the lack of knowledge how IDO1 affects disease processes, no clinical trials of IDO1-targeted therapies of sepsis have been conducted yet. In contrast to the prevention, we demonstrated in this study IDO1 targeting alone failed to save septic animals if 1-MT was administrated after sepsis initiation, however, the combination of 1-MT and tryptophan supplementation significantly improved outcomes of animal sepsis. Due to the fact that sepsis response quickly deletes tryptophan, our observations call attentions to the tryptophan restoration when designing IDO1 targeting-based clinical interventions of sepsis. However, the translational significance of this combination therapy needs to be carefully evaluated by further studies to uncover the underlying mechanism.

In summary, while most studies of inflammation are focused on the early phase of cytokine storm and its anti-inflammatory drug development, we revealed a NAD synthesis-based mechanism that regulates the late phase of inflammation in response to high stress dose. Particularly the importance of nuclear NAD homeostasis was clarified in determining resolvable or refractory inflammation. Our findings of high dose stress-associated persistence of immune suppression support the immunomodulatory therapy concept of severe inflammatory disease sepsis (74, 75). However, considering that the cytokine storm, immune suppression and resolution are three sequential events of inflammatory process, future studies are needed to understand what governs the switch of NAD synthesis pathways in the whole course of inflammation, thereby, a complete picture of inflammation processes can finally be drawn to provide clues to the development of mechanism-based precision treatment and prevention of inflammation diseases such as sepsis, AIDS- or aging- associated severe inflammation upon infections. In addition, because of the limitation of THP-1 cell model, whether the immune tolerance mechanism equally drives the innate and adaptive immune cell tolerance and the universal tolerance noted in failing organs during severe inflammation cannot be concluded from this study but warrants further researches in animal models.

## Data Availability Statement

All datasets generated for this study are included in the manuscript/Supplementary Files.

## Ethics Statement

Human venous blood samples of healthy volunteers or sepsis patients were collected after obtaining written, informed consent, and according to the protocol approved by the Ethics Committee of Shanghai Public Health Clinical Center, Fudan University.

## Author Contributions

JZ, JT, and YL equally contributed to this study. LX assisted experiments. FL and MW recruited and collected clinical samples. SZ directed CRISPR-Cas9-based gene knockout. TL conceived and designed this study, performed data analysis and interpretation, and writing the manuscript. XZ and CM reviewed and discussed the manuscript.

### Conflict of Interest

The authors declare that the research was conducted in the absence of any commercial or financial relationships that could be construed as a potential conflict of interest.
