# Supplementary figures and images for: Switch of NAD Salvage to de novo Biosynthesis Sustains SIRT1-RelB-Dependent Inflammatory Tolerance
Source: Front Immunol. 2019 Oct 11;10:2358. doi: 10.3389/fimmu.2019.02358 (PMC6797595; doi:10.3389/fimmu.2019.02358)

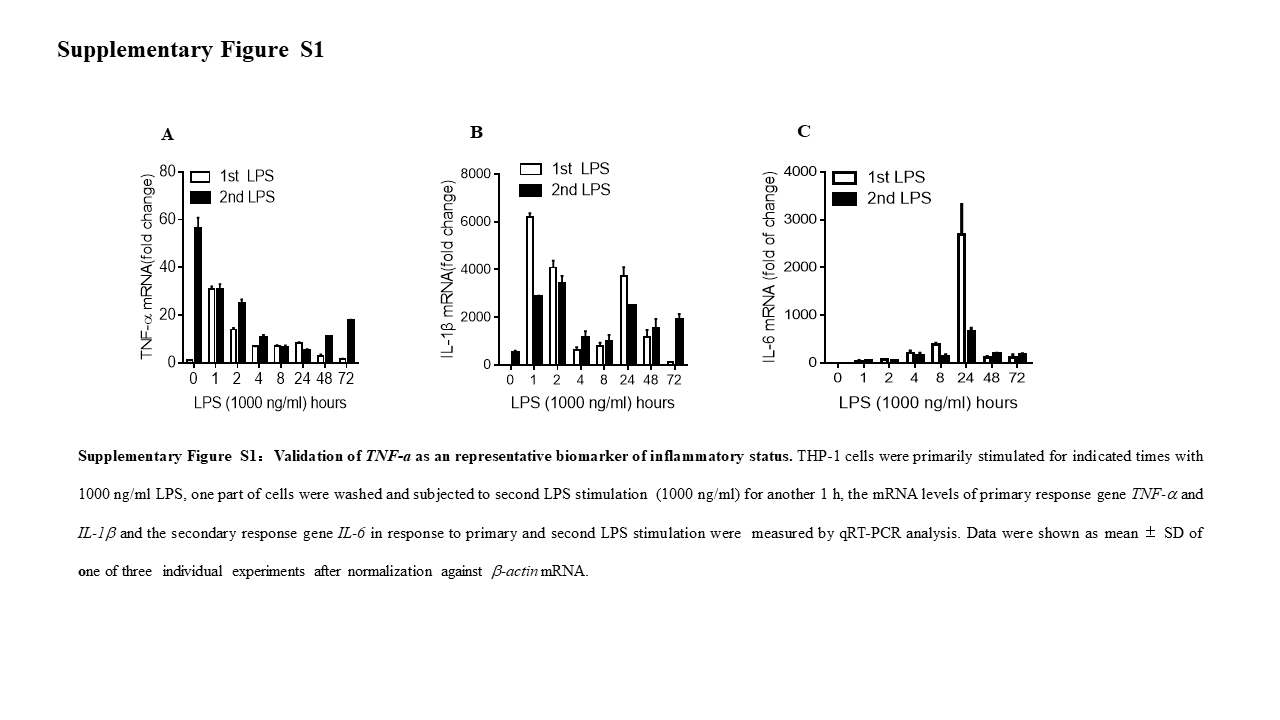

Supplement: Supplementary file 1 [file Image_1.TIF]

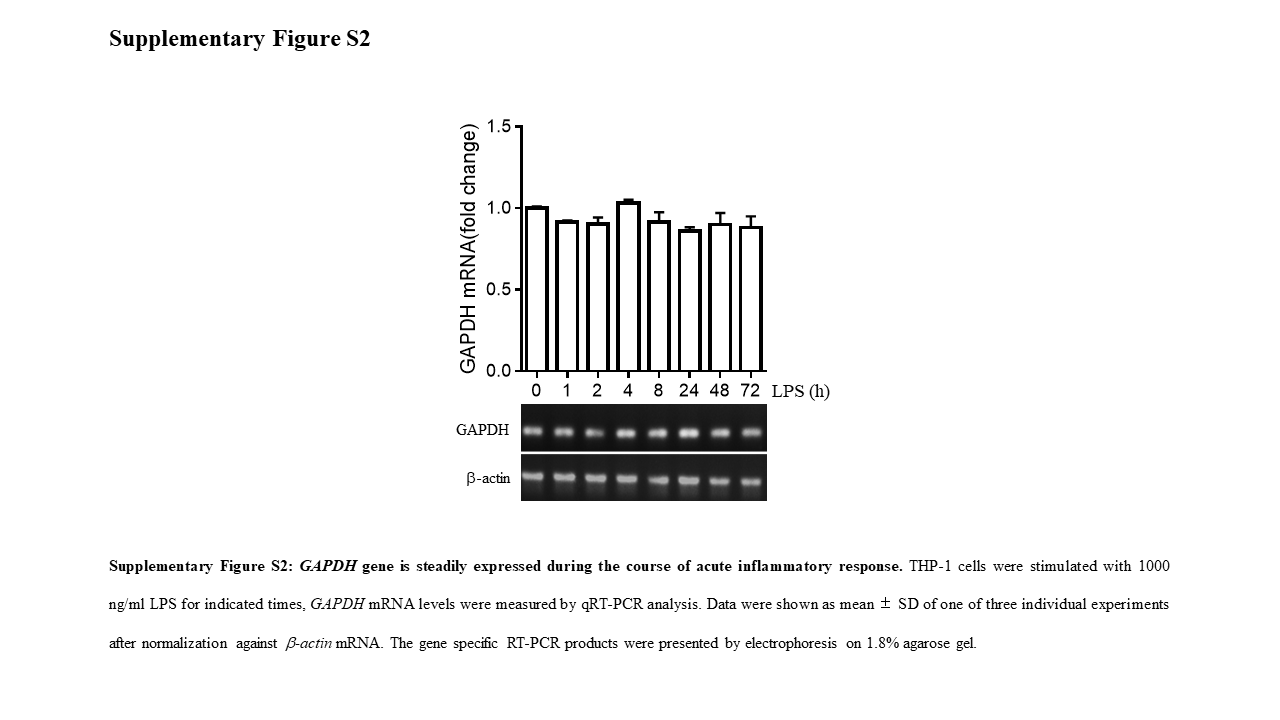

Supplement: Supplementary file 2 [file Image_2.TIF]

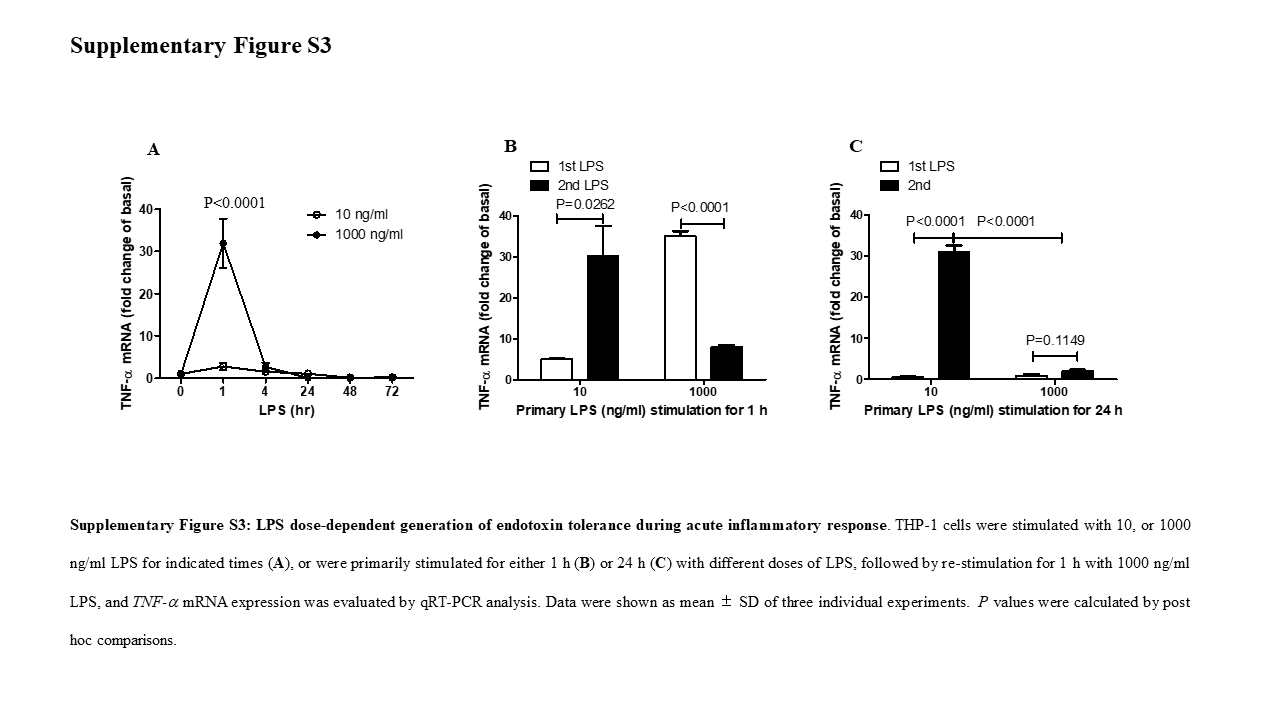

Supplement: Supplementary file 3 [file Image_3.TIF]

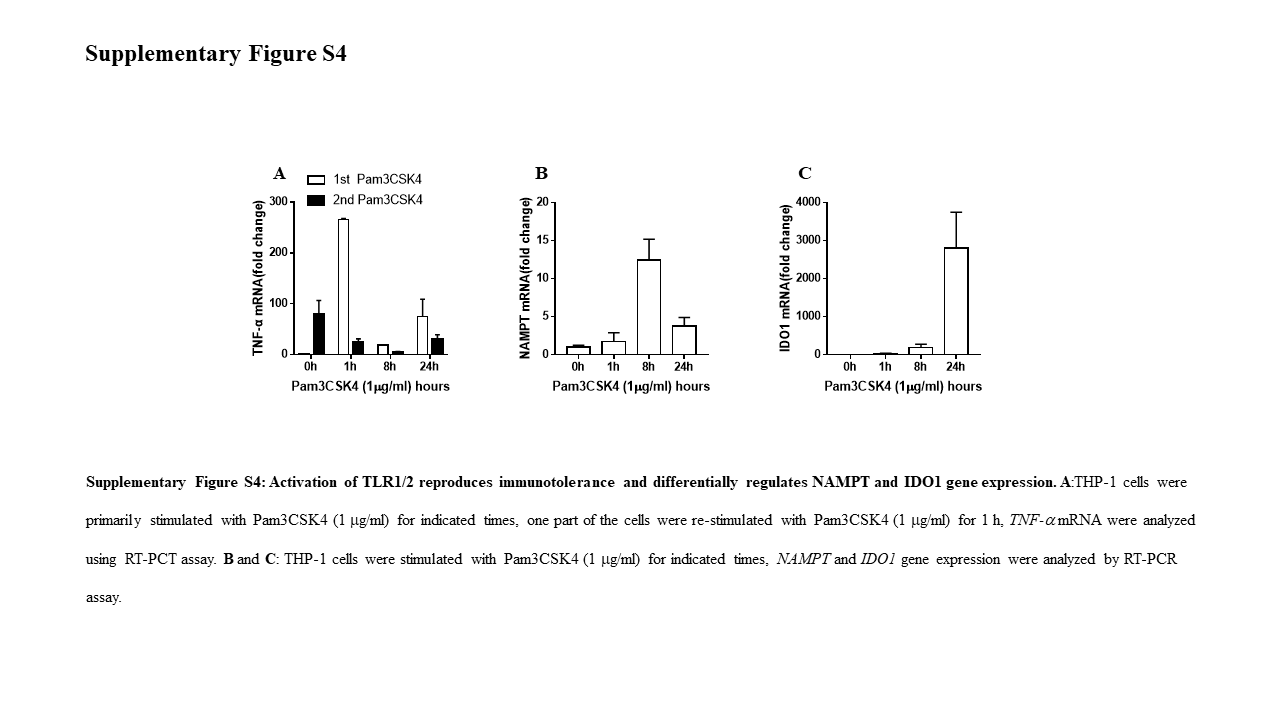

Supplement: Supplementary file 4 [file Image_4.TIF]

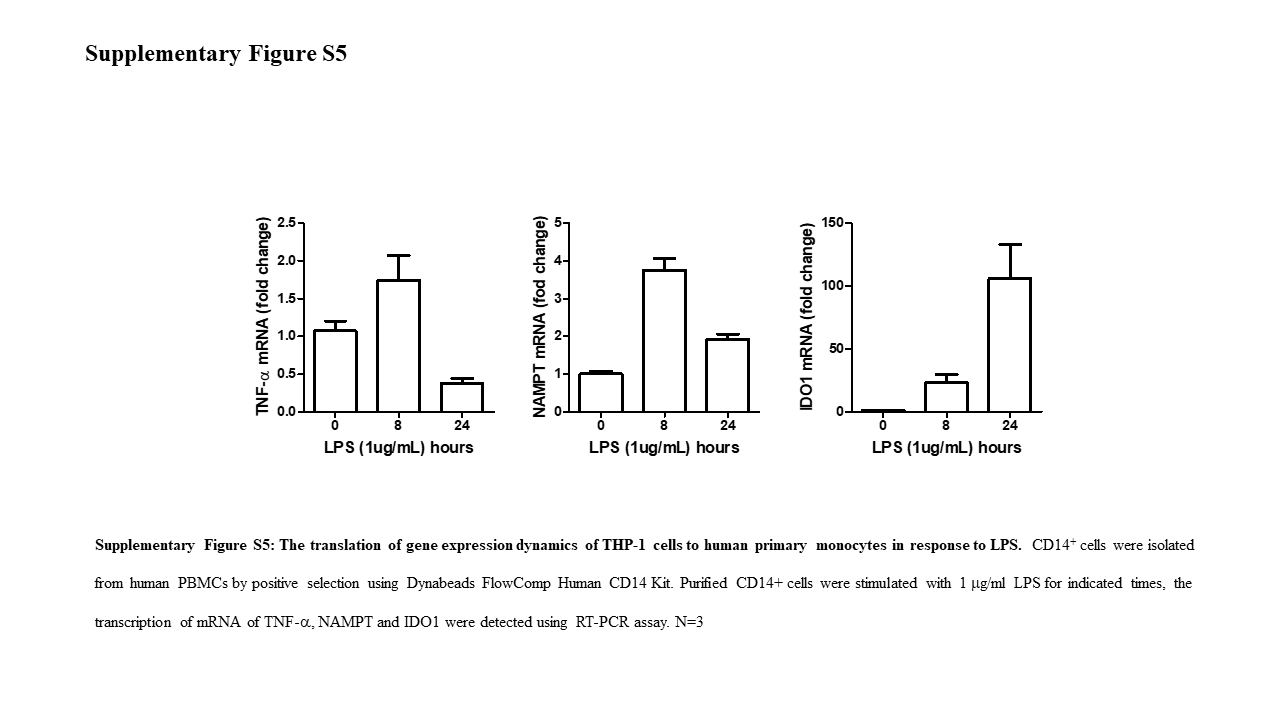

Supplement: Supplementary file 5 [file Image_5.TIF]
